# Comparative lipidomic analysis of phospholipids of hydrocorals and corals from tropical and cold-water regions

**DOI:** 10.1371/journal.pone.0215759

**Published:** 2019-04-29

**Authors:** Andrey B. Imbs, Ly P. T. Dang, Kien B. Nguyen

**Affiliations:** 1 National Scientific Center of Marine Biology, Far Eastern Branch, Russian Academy of Sciences, Vladivostok, Russian Federation; 2 Institute of Natural Products Chemistry, Vietnam Academy of Science and Technology, Hanoi, Vietnam; 3 Graduate University Science and Technology, Vietnam Academy of Science and Technology, Hanoi, Vietnam; 4 Soils and Fertilizers Research Institute, Vietnam Academy of Agricultural Sciences, Hanoi, Vietnam; University of Florida, UNITED STATES

## Abstract

To expand our knowledge of lipid and fatty acid (FA) biosynthesis in marine cnidarians, polar lipidomes of hydrocorals were studied for the first time and then compared with those of soft corals from tropical and boreal regions. The structure and content of FAs and molecular species of ethanolamine, choline, serine, and inositol glycerophospholipids (PE, PC, PS, and PI, respectively), and ceramide aminoethylphosphonate (CAEP) in tropical hydrocorals (*Millepora platyphylla*, *M*. *dichotoma*) and the cold-water hydrocoral *Allopora steinegeri* were determined by chromatography and mass spectrometry. All soft corals and cold-water hydrocorals are characterized by a considerable amount of C_20_ polyunsaturated FAs (PUFAs) elongated into C_22_ PUFAs. In the *Millepora* species, the high level of 22:5n-6 and 22:6n-3 against the background of the extremely low level of C_20_ PUFAs may be explained by a high activity of rare Δ4 desaturase. In contrast to hydrocorals, soft corals are able to elongate and further desaturate C_22_ PUFAs into C_24_ PUFAs. *Allopora* and soft corals use C_20_ PUFAs mainly for the synthesis of PE and PC. The molecular species of PS of soft corals concentrate C_24_ PUFAs, while in *Allopora* and *Millepora* the PS molecules are mainly based on 22:4n-6 and 22:5n-6 acyl groups, respectively. Short acyl groups (C_14_) dominate the CAEP molecules of *Allopora*. In all the animals compared, most molecular species of PE and PC are ether lipids, but diacyl molecular species dominate PI. Hydrocorals and tropical soft corals contain diacyl and ether PS molecules, respectively, whereas cold-water soft corals contain a mixture of these PS forms. The high similarity of the alkyl/acyl compositions indicates a possible biosynthetic relationship between PS and PI in hydrocorals. The data obtained in our study will provide a resource to further investigate the lipid metabolism in marine invertebrates.

## Introduction

Corals occur at depths of up to 6 km from polar to tropical waters of the World Ocean. Hard (or reef-building) corals, which have a hard exoskeleton, and soft corals are widely distributed in the tropical zones [1]. Hydrocorals are also members of tropical coral reef ecosystems [2]. For example, “fire coral” *Millepora platyphylla*, is an important component of Indo-Pacific reefs [3]. Hard and soft corals belong to the class Anthozoa (the phylum Cnidaria). Hydrocorals resemble hard corals in appearance but belong to the class Hydrozoa [4]. In tropical regions, most shallow-water hard and soft corals species, as well as *Millepora*, contain endocellular symbiotic dinoflagellates (SDs, microalgae of the family Symbiodiniaceae) referred to as zooxanthellae [5]. SDs are an essential source of photosynthetic organic carbon for their host [6,7]. Many species of hard corals, soft corals, and hydrocorals inhabit the deep-sea and cold-water zones [8,9], but all these cnidarian species do not contain SDs. In warm and cold waters, corals and hydrocorals are an integral part of benthic communities with very high biodiversity [1,10].

In recent decades, a wide range of ecological and biological processes in coral communities have been investigated extensively [11–14]. Lipids and their fatty acids (FAs) are involved in the majority of biochemical and physiological processes in coral polyps [15] and, therefore, frequently applied as biochemical markers and indicators in coral research [16,17]. Up to 30% of polyps’ dry biomass is comprised of lipids [18], which serve as long-term energy stores [19,20]. Total lipid level is used as an overall indicator to estimate the energetic status of coral colonies during the annual cycle [21], spawning [22,23], light regime changes [24], and environmental stresses [25].

Cnidarian total lipids are a large and diverse group of compounds consisting of non-polar storage lipid classes, such as wax esters and triglycerides, and polar structural lipid classes, for example, glycerophospholipids [18,26]. Measuring the levels of different lipid classes provides more detailed information than a determination of total lipids. A balance between storage and structural lipids is important for species-specific thermal resistance of corals [27] and for depth adaptation of cnidarians [28]. A ratio of lipid classes in coral colonies may be a critical factor for surviving a bleaching event [25] and indicates a supply from SDs [29,30]. The lipid class composition of corals and hydrocorals is related to their taxonomic position and geographic region [29].

Both storage and structural lipid classes mentioned above are esters of fatty acids (FAs). There are about 30 major FAs esterified the lipids of corals and hydrocorals [18]. Today, FAs are the most flexible markers among lipid indicators suitable for a study of coral communities. The application of acyl FAs for chemotaxonomy of corals and hydrocorals has been demonstrated [29,31,32]. The unique FA markers of SDs and host tissues of soft corals and hydrocorals have been found [29,33,34]. The composition of acyl FAs is used to identify major food sources and trophic relationships of corals [16,20,35,36], determine thermal sensitivity and stress of SDs [37,38], and confirm the interchange of lipids between coral host and their SDs [34,39,40].

To date, total lipids, lipid classes, and FAs constitute a basis for lipid studies of marine cnidarians. In fact, FAs are obtained by chemical decomposition of a mixture of thousands of lipid molecules, as well as each lipid class is a mixture of hundreds of lipid molecular species [41]. A set of all lipid molecular species present in an organism is defined as lipidome, and the quantitative description of the lipidome is one of the main tasks of lipidomics. Lipidomic analyses started in the early 2000s and have recently been applied to mollusks [42,43], sea anemones [44], hard corals [45], soft corals and their SDs [46,47], but data on lipidomes of marine invertebrates still remain very limited. In addition to common analyses of lipids, the lipidomic approach opens up new opportunities for marine ecological studies [44]. A comparison of lipid molecular species from the different taxonomic groups provides new knowledge of cnidarian lipid biochemistry [46]. Polar structural lipids, such as phosphono- and phospholipids, are a relatively conserved part of lipidome and are influenced by the environment and food sources to a much lesser extent than non-polar storage lipids [17]. In this respect, a polar part of the cnidarian lipidome seems to be most informative for studies of the chemotaxonomic characteristics of corals and hydrocorals.

To date, the polar lipidomes of three tropical soft corals (*Xenia* sp., *Capnella* sp., and *Sinularia macropodia*) and two cold-water soft coral (*Gersemia rubiformis*, *G*. *fruticosa*) [46,48–50], as well as the glycerophosphocholine molecular species profile of the tropical hard coral *Seriatopora caliendrum* [45], have been described. Information on lipids of hydrocorals is much less than on coral lipids. The polar lipidome of any hydrocoral species was not determined. In the present study, the lipid class and FA compositions of two tropical hydrocorals (*Millepora platyphylla* and *M*. *dichotoma*) and the cold-water hydrocoral *Allopora steinegeri* were compared and the role of Δ4 desaturase in hydrocoral FA biosynthesis was discussed. The chemical structure and composition of polar lipid molecular species of these hydrocoral species were determined for the first time by high-performance liquid chromatography (HPLC) with high-resolution tandem mass spectrometry (MS/MS). To highlight the features of the acyl group distribution, head-group exchange and biosynthesis of phospholipids in cnidarians, the polar lipidomes of hydrocorals were compared with those of soft corals from tropical and cold-water regions.

## Materials and methods

### Chemicals

All solvents were of HPLC or liquid chromatography–mass spectrometry (LC–MS) grade. Polar lipid standards (16:0–20:4 PC, 16:0–20:4 PE, 16:0–20:4 PS, 18:0–20:4 PI, C18(Plasm)-20:4 PC, C18(Plasm)-20:4 PE, C16-18:1 PC) were purchased from Avanti Polar Lipids Co. (Alabaster, USA). Neutral lipid standards (stearyl oleate, cholesterol oleate, 1-*O*-hexadecyl-2,3-dihexadecanoyl-rac-glycerol, glycerol trioleate, oleic acid, cholesterol) and column silica gel (high-purity grade, 70–230 mesh) were from Sigma-Aldrich Co. (St. Louis, USA). A mixture of PUFA methyl esters No. 3 from menhaden oil was obtained from Supelco (Bellefonte, USA). 2-Amino-2-methyl-1-propanol and trifluoroacetic anhydride (Fluka, Germany) were used for the synthesis of 4,4-dimethyloxazoline (DMOX) derivatives of FAs. The precoated silica gel thin-layer chromatography (TLC) plates with a silica sol binder on aluminum foil (PTLC-AF-V) were provided by Sorbfil (Krasnodar, Russian Federation).

### Collection of specimens

Colonies of the hydrocoral *Allopora steinegeri* Fisher, 1938 (Cnidaria: Hydrozoa: Anthoathecata: Stylasteridae) were collected by dredge near the Urup Island (the Kurile Islands, 45°34' N, 149°56' E) at a depth of 200 m (temperature of 1.8°C, salinity of 33.7‰). Colonies of the hydrocoral *Millepora platyphylla* Hemprich & Ehrenberg, 1834 and *Millepora dichotoma* Forskål, 1775 (Cnidaria: Hydrozoa: Anthoathecata: Milleporidae) were collected by SCUBA near the Re Island (the South China Sea, 15°23' N, 109°06' E) at a depth of 4 m (temperature of 28°C, salinity of 32.3‰). Three colonies of each hydrocoral species were used for lipid analysis. The colonies were carefully cleaned of all noncoral debris.

### Lipid analysis

#### Lipid extraction

Total lipids were extracted from fresh animal samples immediately aboard the vessel. To avoid a contamination from plastic, only glass labware was used for lipid extraction. The extraction technique by Folch et al. [51] was modified. The animal samples were crushed and homogenized in a chloroform:methanol (1:2, by vol.) mixture (30 mL for 10 g of wet weight). The obtained homogenate was filtered through the ash-free prewashed paper filter (5–8 μm), and the residue was repeatedly extracted (6 h, 4°C) in a chloroform:methanol (2:1, by vol.) mixture (2 × 30 mL) and filtered. The extracts were then mixed and separated into two layers by adding 35 mL of water and 30 mL of chloroform. The lower layer was collected and evaporated under nitrogen at 40°C with a rotary evaporator IKA RV8 equipped with a chiller IKA RC2control, a water bath IKA HB10 (IKA Werke, Staufen, Germany), and a vacuum pumping unit Vacuubrand PC 201 NT (Wertheim, Germany). The total lipids were dissolved in chloroform and stored at −80°C.

#### Lipid class analysis

According to [52], polar lipids (PL) were isolated from the total lipids by low pressure liquid chromatography on a column with silica gel. In brief, the column was sequentially washed with chloroform and acetone, and then the PL fraction was eluted with methanol with 5% water. Lipid classes were separated by one-dimensional TLC. Each sample was placed on two TLC plates (10 cm × 10 cm). For total lipid analysis, one plate was first developed to its full length with hexane:diethyl ether:acetic acid (70:30:1 by vol.) and finally to 25% length with chloroform: MeOH:28% NH_4_OH (65:35:5 by vol.). For PL analysis, the other plate was developed with the latter solvent system. After drying in air stream, plates were sprayed with 10% H_2_SO_4_/MeOH and heated at 240°C for 10 min. The chromatograms were scanned with an image scanner Epson Perfection 2400 PHOTO (Nagano, Japan) in a grayscale mode. Percentage of lipid content was calculated based on the band intensity using an image analysis program Sorbfil TLC Videodensitometer (Krasnodar, Russia). Units were calibrated with the use of standards for each lipid class as described previously [28].

#### Fatty acid analysis

Fatty acid methyl esters (FAME) were obtained by treating the lipids with 2% H_2_SO_4_/MeOH at 80°C for 2 h in a screw-caped vial under argon, extracted with hexane and purified by preparative TLC developed in benzene. 4,4-Dimethyloxazoline (DMOX) derivatives of FAs were prepared according to [53]. A gas chromatography analysis of FAME was conducted on a GC-2010 chromatograph (Shimadzu, Kyoto, Japan) with a flame ionization detector. A SUPELCOWAX 10 (Supelco, Bellefonte, PA) capillary column (30 m × 0.25 mm i.d.) was used at 210°C. The injector and detector temperatures were 240°C. Helium was used as the carrier gas at a linear velocity of 30 cm/s. The identification of FAs was confirmed by gas chromatography–mass spectrometry (GC–MS) of their methyl esters and DMOX derivatives using a GCMS-2010 Ultra instrument (Shimadzu, Kyoto, Japan) (electron impact at 70 eV) and a MDN-5s (Supelco, Bellefonte, PA) capillary column (30 m × 0.25 mm ID). The carrier gas was He at 30 cm/s. The GC–MS analysis of FAME was performed at 160°C with a 2°C/min ramp to 240°C that was held for 20 min. The injector and detector temperatures were 250°C. GC–MS of DMOX derivatives was performed at 210°C with a 3°C/min ramp to 270°C that was held for 40 min. The injector and detector temperatures were 270°C. Spectra were compared with the NIST library and FA mass spectra archive [54].

#### Analysis of lipid molecular species

Separation of lipids was performed on a Prominence liquid chromatograph consisted of two LC-20AD pump units, a high pressure gradient forming module, CTO-20A column oven, SIL-20A auto sampler, CBM-20A communications bus module, DGU-20A 3 degasser, and a Shim-Pack diol column (50 mm × 4.6 mm ID, 5 μm particle size) (Shimadzu, Kyoto, Japan). Lipid samples and authentic standards were eluted with a binary gradient of (A) hexane:2-propanol:AcOH:Et_3_N (82:17:1:0.08, by vol) and (B) 2-propanol:H_2_O:AcOH:Et_3_N (85:14:1:0.08, by vol). The gradient started at 5% mixture B, and its percentage increased to 80% over 25 min. This composition was maintained for 1 min, returned to 5% mixture B over 10 min, and maintained at 5% for 4 min (the total run time was 40 min). The flow rate was 0.2 mL/min. The column was kept at 40°C. The injection volume was 10 μL. The eluent outlet was connected to a MS analyzer. Lipids were detected by a high resolution tandem (ion trap–time of flight) mass spectrometry on a Shimadzu LCMS-IT-TOF instrument (Kyoto, Japan) operating both in positive and negative modes during each analysis in electrospray ionization (ESI) conditions. The ion source temperature was 200°C, the range of detection was *m/z* 100–1200, and the potential in the ion source was − 3.5 and 4.5 kV for negative and positive modes, respectively. The drying gas (N_2_) pressure was 200 kPa. The nebulizer gas (N_2_) flow was 1.5 L/min. MS^2^ was used consistently, and MS^3^ was applied for PC molecular species. For MS/MS analysis, spectra were obtained scanning from *m/z* 100–1200 at one cycle (MS(+/−) and MS/MS(+/−)) per 1.5 s with an isolation width of 3 Da. According to the recommendations of the manufacturer, the collision energy was 50% of the frame, Ar was the collision gas (50% of the frame), ion accumulation time was 10 ms. The instrument was calibrated using Shimadzu tuning mix. The mass accuracy was between 2 and 7 ppm. PL molecular species were identified as described earlier [48]. Quantification of individual molecular species within each PL class was carried out by calculating the peak areas for the individual extracted ion chromatograms [55].

### Statistical analysis

Significance of differences in pairs of mean contents of FAs and lipid molecular species between hydrocorals was tested by one-way analysis of variance (ANOVA). The raw data were used following evaluation of the homogeneity of variances (Leven’s test) and the normality of data distribution (Shapiro–Wilk test). All statistical analyses were performed using STATISTICA 5.1 (StatSoft, Inc., USA). A statistical probability of *p* < 0.01 was considered significant. Values are reported as the mean ± standard deviation.

### Ethical approval

This article does not contain any studies with human participants or vertebrate animals performed by any of the authors. All the experiments on invertebrate animals were reviewed and approved by the Ethics Committee of the National Scientific Center of Marine Biology of the Far Eastern Branch of the Russian Academy of Sciences and conducted in agreement with the principles expressed in the Declaration of Helsinki.

## Results

### Composition of lipid classes and fatty acids of hydrocorals

The composition of total lipids (TL) and polar lipids (PL), as well as their FA profiles, of two tropical hydrocorals (*M*. *platyphylla*, *M*. *dichotoma*) and the cold-water hydrocoral *A*. *steinegeri* were analyzed by TLC, GC, and GC–MS. The hydrocoral TL contained 15–26% PL, which comprised five major classes: ethanolamine glycerophospholipids (PE), choline glycerophospholipids (PC), serine glycerophospholipids (PS), inositol glycerophospholipids (PI), and ceramide aminoethylphosphonate (CAEP), with the dominance of PC and PE (S1 and S2 Tables).

**Fig 1:**
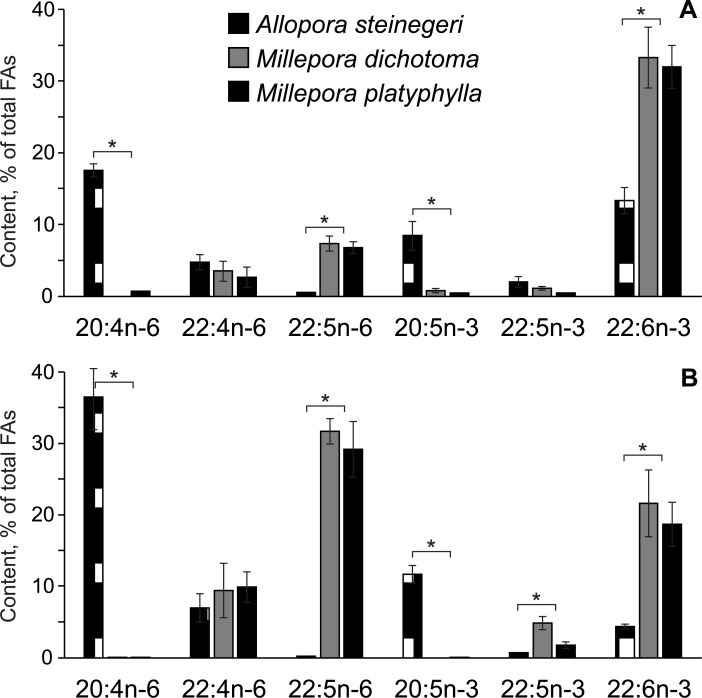
Comparison of the content of the polyunsaturated fatty acids in hydrocoral lipids. The level of C_20-22_ polyunsaturated fatty acids (% of total acids, mean ± SD, *n* = 3) obtained from (A) total lipids and (B) polar lipids of the hydrocorals *Allopora steinegeri* (dotted bars), *Millepora dichotoma* (gray bars), and *M*. *platyphylla* (black bars). Acids of n-6 series are as follows: 20:4n-6, arachidonic; 22:4n-6, docosatetraenoic; 22:5n-6, docosapentaenoic. Acids of n-3 series are as follows: 20:5n-3, eicosapentaenoic; 22:5n-3, docosapentaenoic; 22:6n-3, docosahexaenoic. (*) Significant difference (*p* < 0.01) between *Allopora* and *Millepora*.

The characteristic unsaturated FAs of *A*. *steinegeri* were eicosenoic (20:1n-9), arachidonic (20:4n-6), eicosapentaenoic (20:5n-3), and docosahexaenoic (22:6n-3) acids. Both *Millepora* species contained insignificant amounts of C_20_ unsaturated FAs and an extremely high content of 22:6n-3 (up to 33% of total FAs) (S1 and S3 Tables, Fig 1A). A significantly higher level of 22:6n-3 (ANOVA test, *p* < 0.01) was found in both TL and PL of *Millepora*, when compared to *Allopora* (Fig 1).

Two 22:5 isomers were found in hydrocoral lipids. We placed special emphasis on the chemical structure of n-6 and n-3 isomers of 22:5 acid. The positions of double bonds in the isomers were confirmed by mass spectrometry of their 4,4-dimethyloxazoline (DMOX) derivatives (S1 Fig). The substantial amount of 22:5n-6 in the *Millepora* species distinguishes them from *A*. *steinegeri* (Fig 1A). Moreover, 22:5n-6 was the major polyunsaturated FA (PUFA) obtained by hydrolysis of PL of *Millepora* (about 30% of total FAs) (Fig 1B). The 22:5n-3 acid was also detected in *Millepora* but the average percentage of this isomer was ten-fold lower than that of 22:5n-6 (Fig 1B). In contrast to *Millepora*, 22:5n-6 was practically absent in acyl groups of PL molecules of *Allopora* (Fig 1B). In PL molecules of *Allopora*, the major C_22_ PUFA of n-6 series was 22:4n-6 (Fig 1B). This acid was also detected in *Millepora*. There was no difference in the level of 22:4n-6 between *Millepora* and *Allopora* lipids (ANOVA test, *p* > 0.05) (Fig 1).

### Molecular species of phosphonolipids of hydrocorals

**Fig 2:**
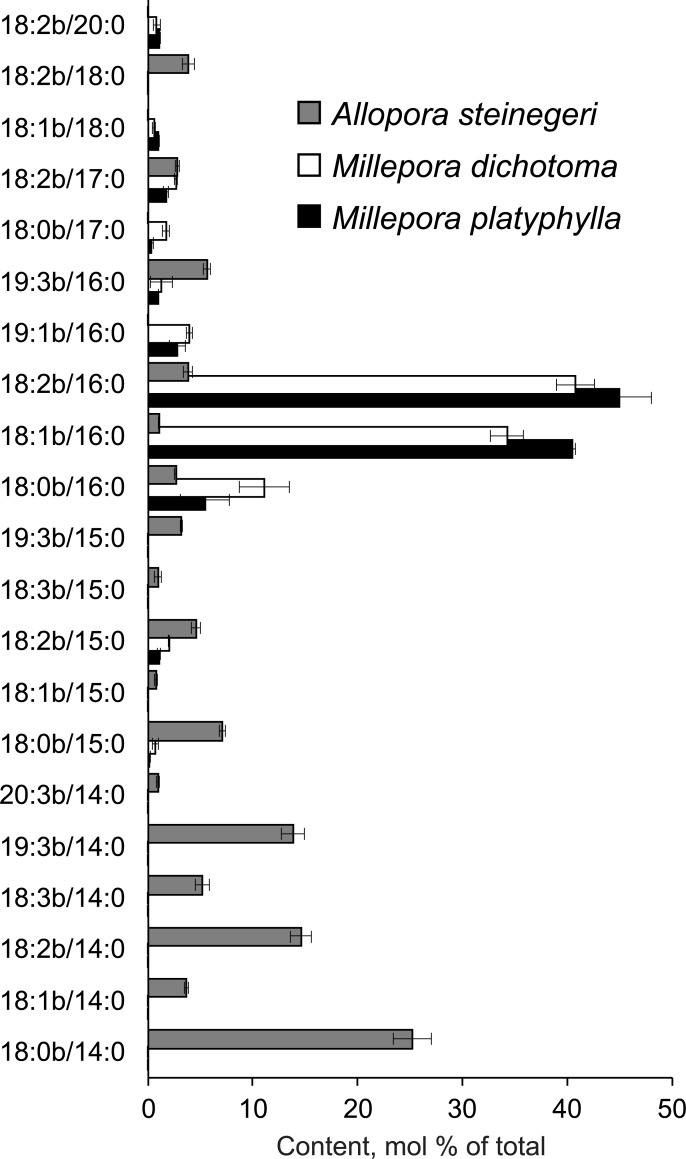
Comparison of ceramide aminoethylphosphonate (CAEP) profiles of hydrocorals. Content of CAEP molecular species (mol % of total CAEP, mean ± SE, *n* = 3) obtained from lipids of the hydrocorals *Allopora steinegeri* (grey bars), *Millepora dichotoma* (white bars), and *M*. *platyphylla* (black bars).

The content of CAEP in total lipids of *Millepora* (2.2–3.0%) were close to that of *Allopora* (3.0%) (calculated from S2 Table), but these hydrocoral genera had different CAEP compositions. In the hydrocorals studied, a total of 21 molecular species of CAEP were detected; in particular, 11 and 17 molecular species were found in *Millepora* and *Allopora*, respectively (S4 Table). The molecular species with palmitic acid (16:0) linked to the monoenoic and dienoic sphingoid bases comprised up to 85% of total CAEP of *Millepora*, while myristic acid (14:0) derivatives formed a major part of CAEP molecular species in *Allopora* (Fig 2). In *Millepora*, 18:2b/16:0 CAEP and 18:1b/16:0 CAEP accounted for 6.38−6.51 and 5.36−5.86% of total polar lipids, respectively. The main CAEP molecular species of *Allopora* was 18:0b/14:0 CAEP (2.95% of total polar lipids). In addition to sphingosine and sphingadiene (18:1b and 18:2b), ceramide components containing other dihydroxy bases such as 18:0b, 18:3b, 19:1b, 19:3b, and 20:3b were also detected. The 15:0, 17:0, 18:0, and 20:0 acids were also found in the hydrocoral CAEP. The specimens of *Allopora* contained a significantly higher level of CAEP molecular species with odd-chain *N*-acyl groups than the specimens of *Millepora* (ANOVA test, *p* < 0.01).

### Molecular species of glycerophospholipids (GPL) of hydrocorals

The chemical structure and content of the molecular species of glycerophospholipids (GPL) from hydrocorals were analyzed by a high-resolution MS/MS with the special attention to the distribution of 22:4 and 22:5 acyl groups. Diacyl GPL molecular species are abbreviated as X/Y with X and Y acyl groups presumably in *sn*-1 and *sn*-2 positions, respectively. Alkylacyl GPL molecular species are abbreviated as Xe/Y with X, alkyl group, and Y, acyl group.

**Fig 3:**
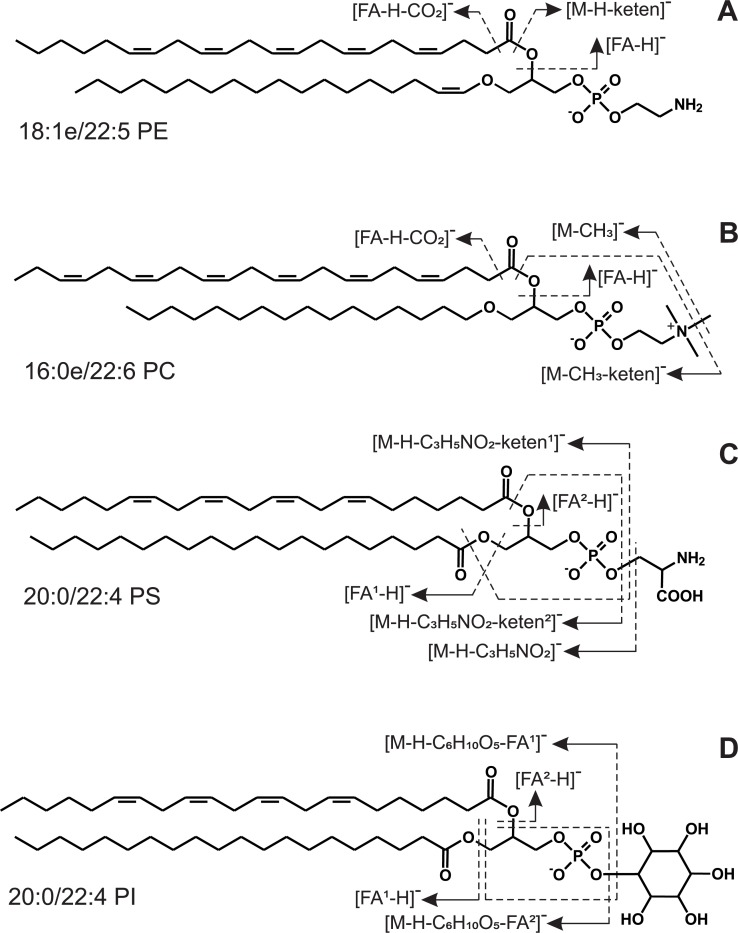
Mass spectrometric fragmentation of glycerophospholipids (GPL) from hydrocorals. MS/MS fragmentation patterns of negative ([M−H]^−^) molecular ions of four GPL classes exemplified by molecules of (A) ethanolamine glycerophospholipid (18:1e/22:5 PE), (B) choline glycerophospholipid (16:0e/22:6 PC), (C) serine glycerophospholipid (20:0/22:4 PS), and (D) inositol glycerophospholipid (20:0/22:4 PI). These GLP molecular species were found in the hydrocorals *Allopora steinegeri*, *Millepora dichotoma*, and *M*. *platyphylla*.

The brief MS/MS fragmentation pattern of molecular ions of each GLP class (PE, PC, PS, and PI) is shown in Fig 3. Each PE molecular species produced [M+H]^+^, [M+H+Et_3_N]^+^, and [M−H]^−^ ions. The MS^2^ spectrum of [M−H]^−^ ions of PE molecule contained three major peaks ([M−H−keten]^−^, [FA−H]^−^, and [FA−H−CO_2_]^−^) (Fig 3A). Each PC molecular species produced [M+H]^+^, [M+HCOO]^−^, and [M−CH_3_]^−^ ions. The characteristic ions of the MS^2^ spectrum of the [M−CH_3_]^−^ were [M−CH_3_−keten]^−^, [FA−H]^−^, and [FA−H−CO_2_]^−^ (Fig 3B). The MS^2^ spectra of PE or PC with two different acyl groups contained two different [FA−H]^−^ ions. The molecular species of diacyl PS were identified by MS^2^ spectra of their [M−H]^−^ ions. These spectra contained several peaks of the characteristic ions: [M−H−C_3_H_5_NO_2_]^−^, [M−H−C_3_H_5_NO_2_−keten^1^)]^−^, [M−H−C_3_H_5_NO_2_−keten^2^]^−^, [M−H−C_3_H_5_NO_2_−FA^2^]^−^, [FA^1^ −H]^−^, and [FA^2^ −H]^−^ (Fig 3C). The MS^2^ spectra of the [M−H]^−^ ions of diacyl PI molecules were also complex and contained at least six diagnostic peeks ([M−H−keten^2^]^−^, [M−H−FA^2^]^−^, [M−H−C_6_H_10_O_5_−FA^1^]^−^, [M−H−C_6_H_10_O_5_−FA^2^]^−^, [FA^1^ −H]^−^, and [FA^2^ −H]^−^ (Fig 3D). The spectra and fragmentations of the major GLP classes were discussed in detail in our previous publications [46,48,49].

The composition of GPL molecular species of the *Millepora* hydrocorals is shown in Table 1. A total of 62 molecular species of PE, PC, PS, and PI were identified. Eight molecular species constituting more than 3% accounted for 60% of total polar lipids: 16:1e/22:5 PE, 18:1e/22:5 PE, 16:0e/22:6 PC, 16:0e/22:5 PC, 16:0/22:6 PC, 18:0e/22:5 PC, 20:0/22:4 PS, and 20:0/22:4 PI. The GLP profiles of two *Millepora* species resembled each other closely.

**Table 1:** Composition of glycerophospholipids from the hydrocorals *Millepora platyphylla* and *M*. *dichotoma*.

Molecular species	[M–H]^–^, *m/z*^*a*^			Content, mol %^*b*^	
	Detected	Calculated	Diff^*c*^	*M*. *platyphylla*	*M*. *dichotoma*
Ethanolamine glycerophospholipids (PE)					
18:1e/17:1^*d*^	714.5442	714.5443	-0.1	0.90 ± 0.18	-
17:1/19:1	742.5371	742.5392	-2.1	0.42 ± 0.03	-
16:1e/22:5	748.5287	748.5286	0.1	2.36 ± 0.36	4.29 ± 0.29
16:0e/22:5	750.5405	750.5443	-3.8	1.13 ± 0.19	1.79 ± 0.21
17:1e/22:5	762.5413	762.5443	-3.0	0.41 ± 0.05	0.27 ± 0.00
18:1e/22:6	774.5410	774.5443	-3.3	0.50 ± 0.06	0.78 ± 0.03
18:1e/22:5	776.5581	776.5599	-1.8	8.59 ± 0.10	8.10 ± 0.12
19:1e/22:5	790.5720	790.5756	-3.6	0.51 ± 0.07	0.23 ± 0.07
18:1/22:5^e^	790.5370	790.5392	-2.2	0.48 ± 0.14	0.41 ± 0.13
18:1/22:5	790.5344	790.5392	-4.8	0.24 ± 0.05	0.95 ± 0.29
18:0/22:6	790.5422	790.5392	3.0	0.11 ± 0.00	0.35 ± 0.15
18:0/22:5	792.5549	792.5549	0.0	0.16 ± 0.04	0.10 ± 0.02
18:0/22:4	794.5648	794.5705	-5.7	0.47 ± 0.08	0.51 ± 0.05
19:1/22:5	804.5491	804.5549	-5.8	3.10 ± 0.48	1.78 ± 0.37
20:1/22:5	818.5663	818.5705	-4.2	1.92 ± 0.52	0.91 ± 0.19
20:0/22:4	822.5940	822.6018	-7.8	0.38 ± 0.03	0.64 ± 0.04
Choline glycerophospholipids (PC)					
15:1e/22:5	778.5818	778.5745	7.3	0.21 ± 0.03	0.34 ± 0.02
16:0e/22:6	792.5876	792.5902	-2.6	12.32 ± 0.11	13.54 ± 0.15
16:0e/22:5	794.6037	794.6058	-2.1	12.69 ± 1.08	12.13 ± 0.53
16:0/22:6	806.5693	806.5695	-0.2	4.03 ± 0.44	3.94 ± 0.13
16:0/22:5	808.5829	808.5851	-2.2	2.57 ± 0.07	1.96 ± 0.09
18:0e/22:6	820.6146	820.6215	-6.9	2.29 ± 0.30	3.21 ± 0.02
18:0e/22:5	822.6321	822.6371	-5.0	3.59 ± 0.60	3.95 ± 0.32
18:1/22:6	832.5787	832.5851	-6.4	0.52 ± 0.06	0.59 ± 0.03
18:0/22:6	834.5922	834.6008	-8.6	1.48 ± 0.17	1.75 ± 0.10
Serine glycerophospholipids (PS)					
18:0/22:4	838.5530	838.5603	-7.3	0.49 ± 0.11	0.45 ± 0.12
19:0/22:4	852.5717	852.5760	-4.3	0.23 ± 0.01	0.18 ± 0.02
20:0/22:5	864.5698	864.5760	-6.2	1.71 ± 0.02	1.11 ± 0.07
20:0/22:4	866.5852	866.5916	-6.4	7.74 ± 0.10	7.83 ± 0.23
21:0/22:4	880.6013	880.6073	-6.0	0.24 ± 0.02	0.26 ± 0.01
Inositol glycerophospholipids (PI)					
16:0e/16:0	795.5340	795.5393	-5.3	0.07 ± 0.00	0.02 ± 0.01
16:0e/17:1	807.5343	807.5393	-5.0	0.07 ± 0.00	0.01 ± 0.01
18:0e/16:0	823.5662	823.5706	-4.4	-	0.04 ± 0.01
16:0/18:2	833.5138	833.5185	-4.7	0.04 ± 0.01	0.00 ± 0.00
18:0e/17:1	835.5691	835.5706	-1.5	0.29 ± 0.02	0.14 ± 0.01
16:0/20:4	857.5154	857.5185	-3.1	0.03 ± 0.01	-
16:0e/22:5	869.5544	869.5549	-0.5	0.25 ± 0.01	0.16 ± 0.01
16:0/22:6	881.5140	881.5185	-4.5	0.43 ± 0.04	0.78 ± 0.03
18:0/20:5	883.5282	883.5342	-6.0	0.13 ± 0.02	-
18:0/20:4	885.5450	885.5498	-4.8	0.14 ± 0.00	0.00 ± 0.00
18:0e/22:5	897.5815	897.5862	-4.7	0.22 ± 0.01	0.19 ± 0.05
18:0e/22:4	899.5961	899.6019	-5.8	0.01 ± 0.01	0.09 ± 0.03
18:1/22:6	907.5277	907.5342	-6.5	0.04 ± 0.01	0.06 ± 0.02
18:0/22:6	909.5436	909.5498	-6.2	0.49 ± 0.01	0.55 ± 0.07
18:0/22:5	911.5589	911.5655	-6.6	0.49 ± 0.01	0.32 ± 0.06
18:0/22:4	913.5750	913.5811	-6.1	1.97 ± 0.02	0.97 ± 0.04
19:0/22:4	927.5918	927.5968	-5.0	0.55 ± 0.02	0.32 ± 0.02
20:0/22:6	937.5736	937.5811	-7.5	0.07 ± 0.01	0.04 ± 0.01
20:0/22:5	939.5932	939.5968	-3.6	0.71 ± 0.02	0.65 ± 0.02
20:0/22:4	941.6083	941.6124	-4.1	7.45 ± 0.14	7.30 ± 0.36
21:0/22:4	955.6212	955.6281	-6.9	0.14 ± 0.01	0.15 ± 0.02

^*a*^[M+H]^+^ for choline glycerophospholipids.

^*b*^Molar percentage of total polar lipids, mean ± SE, *n* = 3.

^*c*^Difference between calculated and measured values of *m/z*, (×10^3^).

^*d*^Structure description; alkyl (alkenyl) chain / acyl chain

^*e*^Structure description; acyl chain / acyl chain

A high diversity of GPL molecules distinguished *Allopora*; a total of 110 molecular species were identified in this cold-water hydrocoral (Tables 2 and 3). The major compounds (reaching more than 3%) were 18:2e/20:4 PE, 20:2e/20:4 PE, 16:0/20:5 PC, 16:0/22:6 PC, 20:1e/20:4 PC, 18:0/22:4 PS, and 18:0/22:4 PI. In contrast to *Millepora*, the group of these major compounds in *Allopora* amounted to less than 30% of total PL.

**Table 2:** Composition of ethanolamine and choline glycerophospholipids (PE and PC) from the hydrocoral *Allopora steinegeri*.

Ethanolamine glycerophospholipids			Choline glycerophospholipids		
Molecular species	[M–H]^–^, *m/z*	Content, mol %^*a*^	Molecular species	[M+H]^+^, *m/z*	Content, mol %
14:1e/20:4^*b*^	694.4775	0.50 ± 0.08	14:1e/20:4	738.5477	0.54 ± 0.04
15:1e/20:4	708.4942	0.14 ± 0.03	14:0e/20:4	740.5653	0.29 ± 0.03
14:0/20:5^c^	708.4558	0.09 ± 0.09	14:0/20:5	752.5256	0.82 ± 0.09
14:0/20:4	710.4729	0.11 ± 0.07	16:1e/20:5	764.5677	0.33 ± 0.02
16:1e/20:5	720.4953	0.42 ± 0.06	14:0e/22:6	764.5677	0.33 ± 0.02
16:2e/20:4	720.4953	0.42 ± 0.06	16:0e/20:5	766.5797	0.42 ± 0.00
16:1e/20:4	722.5098	2.35 ± 0.27	16:1e/20:4	766.5797	1.39 ± 0.00
16:0/20:5	736.4875	2.35 ± 0.57	15:0/20:5	766.5440	0.50 ± 0.03
17:1e/20:4	736.5246	0.25 ± 0.25	16:0e/20:4	768.5997	2.13 ± 0.07
16:0/20:4	738.5038	1.91 ± 0.06	14:0/22:6	778.5407	1.35 ± 0.10
18:2e/20:5	746.5060	0.55 ± 0.08	16:0/20:5	780.5589	3.64 ± 0.03
18:2e/20:4	748.5245	2.90 ± 0.32	18:1e/20:5	792.5900	0.72 ± 0.05
18:1e/20:4	750.5364	2.34 ± 0.02	18:2e/20:4	792.5900	0.72 ± 0.05
17:0/20:5	750.5043	0.15 ± 0.15	15:0/22:6	792.5536	0.60 ± 0.04
18:0e/20:4	752.5522	0.18 ± 0.02	18:1e/20:4	794.6094	2.33 ± 0.18
17:0/20:4	752.5188	0.24 ± 0.01	17:0/20:5	794.5719	0.68 ± 0.13
19:2e/20:4	762.5374	0.72 ± 0.01	17:0/20:4	796.5926	0.74 ± 0.07
18:1/20:5	762.5027	0.60 ± 0.10	16:0/22:6	806.5703	3.06 ± 0.05
16:0/22:5	764.5170	0.32 ± 0.02	18:0/20:5	808.5878	1.66 ± 0.20
18:0/20:5	764.5170	0.55 ± 0.03	18:0/20:4	810.6047	1.30 ± 0.10
18:1/20:4	764.5170	0.69 ± 0.03	20:1e/20:5	820.6186	1.24 ± 0.12
16:0/22:4	766.5323	0.45 ± 0.00	20:2e/20:4	820.6186	0.78 ± 0.08
18:0/20:4	766.5323	1.50 ± 0.01	17:0/22:6	820.5836	0.99 ± 0.06
20:2e/20:5	774.5352	1.24 ± 0.16	20:1e/20:4	822.6389	3.93 ± 0.38
20:2e/20:4	776.5532	6.48 ± 0.93	20:4/20:4	830.5631	0.45 ± 0.03
20:1e/20:4	778.5672	1.59 ± 0.33	18:1/22:6	832.5789	0.61 ± 0.00
21:3e/20:4	788.5519	0.14 ± 0.10	20:1/20:5	834.5979	0.57 ± 0.03
21:2e/20:4	790.5660	0.07 ± 0.06	18:0/22:6	834.5979	0.82 ± 0.05
20:1/20:5	790.5317	0.68 ± 0.01	20:1/20:4	836.6155	0.85 ± 0.04
20:1/20:4	792.5502	0.14 ± 0.06	18:0/22:4	838.6298	0.51 ± 0.01
18:0/22:5	792.5457	0.62 ± 0.02	20:1/22:6	860.6136	0.63 ± 0.00
18:0/22:4	794.5610	0.91 ± 0.05			
22:3e/20:4	802.5667	0.09 ± 0.05			
21:2/20:4	804.5476	1.53 ± 1.18			
21:1/20:4	806.5625	0.10 ± 0.09			
22:2/20:4	818.5655	0.78 ± 0.42			

^*a*^Molar percentage of total polar lipids, mean ± SE, n = 3.

^*b*^Structure description; alkyl (alkenyl) chain / acyl chain

^*c*^Structure description; acyl chain / acyl chain

**Table 3:** Composition of serine and inositol glycerophospholipids (PS and PI) from the hydrocoral *Allopora steinegeri*.

Serine glycerophospholipids			Inositol glycerophospholipids		
Molecular species	[M–H]^–^, *m/z*	Content, mol %^*a*^	Molecular species	[M–H]^–^, *m/z*	Content, mol %
18:0/20:4^*b*^	810.5196	0.71 ± 0.09	18:1e/20:4^*c*^	869.5464	0.15 ± 0.04
18:0/20:1	816.5710	0.17 ± 0.01	18:0e/20:4	871.5692	0.10 ± 0.02
17:0/22:4	824.5376	0.20 ± 0.03	18:1/20:4	883.5343	0.03 ± 0.01
18:0/22:5	836.5351	0.27 ± 0.05	16:0/22:5	883.5343	0.07 ± 0.02
18:0/22:4	838.5509	6.81 ± 0.16	18:0/20:4	885.5569	0.03 ± 0.01
18:0/22:1	844.6047	0.02 ± 0.01	16:0/22:4	885.5569	0.11 ± 0.03
19:1/22:4	850.5533	0.04 ± 0.00	17:0/22:4	899.5625	0.10 ± 0.01
19:0/22:4	852.5689	0.54 ± 0.01	18:1/22:5	909.5414	0.09 ± 0.03
20:1/22:4	864.5662	0.11 ± 0.01	18:0/22:6	909.5414	0.01 ± 0.00
20:0/22:4	866.5945	0.12 ± 0.00	18:0/22:5	911.5579	0.41 ± 0.08
			18:1/22:4	911.5579	0.23 ± 0.04
			18:0/22:4	913.5728	3.92 ± 0.32
			19:1/22:4	925.5737	0.07 ± 0.01
			19:0/22:4	927.5912	0.15 ± 0.02
			20:1/22:5	937.5717	0.03 ± 0.00
			20:1/22:4	939.5933	0.11 ± 0.02

^*a*^Molar percentage of total polar lipids, mean ± SE, n = 3.

^*b*^Structure description; acyl chain / acyl chain

^*c*^Structure description; alkyl (alkenyl) chain / acyl chain

A very uneven distribution of PUFA acyl groups among phospholipids classes was found (Tables 1–3). In all the hydrocoral species studied, 22:4 acyl groups dominated in PS and PI molecular species, whereas trace amounts of this acyl group were detected in PE and PC molecular species. PE and PC of *Allopora* mainly contained the molecular species with 20:4 and 20:5 acyl groups, whereas 22:5 acyl groups were abundant in the same GPL classes of the *Millepora*. In both hydrocoral genera, 22:6 acyl groups concentrated in the molecular species of PC.

Different alkyl/alkenyl compositions of GPL were found in the tropical and cold-water hydrocorals (Tables 1–3). GPL of *Millepora* were characterized by a high level of C_16_ and C_18_ ether groups. A variety of saturated and polyunsaturated alkyl groups (from C_14_ to C_22_ ether groups) were represented in the GPL molecular species of *Allopora*. The level of molecular species with odd-chain alkyl groups was significantly higher in *Allopora* than in *Millepora* (ANOVA test, *p* < 0.01).

### Pairwise comparison of the GPL classes of hydrocorals

As is known, the head-group exchange reaction displaces a polar group of GLP molecules but does not affect the chemical structure of their non-polar parts (acyl/alkylglycerol groups). We supposed that a high similarity between the non-polar parts of two GPL classes may be an indicator of possible head-group exchange and/or joint biosynthetic precursor for these GPL classes. Percentages of the molecular species with the same acyl/alkylglycerol groups were calculated and compared for each pair of phospholipid classes (PE, PC, PS, and PI), (S3 Fig).

A comparison of the acyl/alkylglycerol group composition of the pairs of GPL classes showed a close similarity between PS and PI in all the hydrocoral species (S3 Fig). For example, in *Allopora*, 96.6% of total PS molecular species and 81.8% of total PI molecular species had the same acyl/alkylglycerol groups (S3 Fig). No similarity between PS and PC molecular species was found in the both *Millepora* species. More than a half of the PE and PC molecular species in *Allopora* had the same acyl/alkylglycerol groups. Ambiguous data were obtained for other pairs of GPL classes.

### Comparison of GPL forms of hydrocorals and soft corals

**Fig 4:**
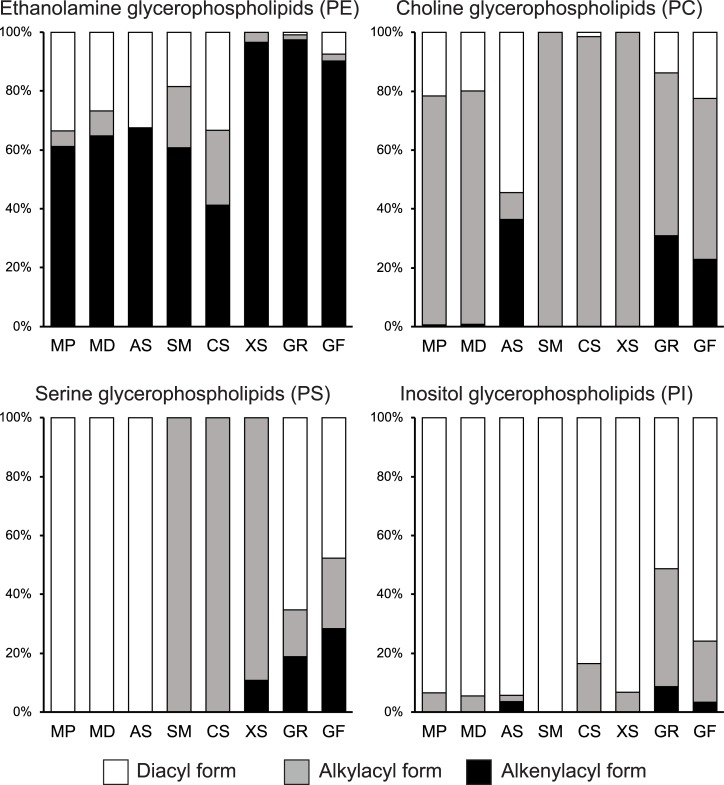
Distribution of the alkenylacyl, alkylacyl, and diacyl forms in each phospholipid class of hydrocoral and soft coral species. Three hydrocoral species were studied: MP, *Millepora platyphylla*; MD, *Millepora dichotoma*; AS, *Allopora steinegeri*. Polar lipidomes of five soft coral species were earlier described: SM, *Sinularia macropodia* [46]; CS, *Capnella* sp. [46]; XS, *Xenia* sp. [48]; GR, *Gersemia rubiformis* [49]; GF, *Gersemia fruticosa* [50].

1-*O*-Alkyl-2-acyl, 1-*O*-alkenyl-2-acyl (plasmalogen), and 1,2-diacyl forms of GPL were identified in the hydrocorals. The proportion of these lipid forms highly varied depending on the phospholipid class. The proportions of hydrocoral GPL forms were compared with those of five soft coral species described earlier (the tropical species: *Sinularia macropodia*, *Capnella* sp., and *Xenia* sp. [46,48]; the cold-water species: *Gersemia fruticosa* and *G*. *rubiformis* [49,50]) (Fig 4). In all cnidarians, the plasmalogen form (40–95% of total) dominated PE molecular species. The alkylacyl form was abundant in PC molecular species of the tropical cnidarians (80–100% of total), whereas PC of the cold-water cnidarians also contained large proportions of the plasmalogen and diacyl forms. Most of PI molecules were detected in the diacyl form.

Clear species-specific differences in the form and composition of PS molecular species were observed between hydrocorals and soft corals (Fig 4). Only the diacyl form was found in PS molecules of hydrocorals. The alkylacyl form dominated PS of tropical soft corals. All three forms were detected in PS of cold-water soft corals. The PS molecular species of these three cnidarian groups also differed in the characteristic acyl groups: 22:4 were found in hydrocorals, C_24_ PUFAs in tropical soft corals, and C_24_ PUFAs + 20:1 in cold-water soft corals (Tables 1–3, ref. [46,49,50]).

## Discussion

### Comparison of possible pathways of PUFA synthesis in cnidarians

PUFAs are essential to the life of animals. The ability to synthesize certain PUFA set is a key characteristic of each animal taxon [56,57]. PUFAs with methylene-interrupted double bonds and 22 carbon atoms in their chains (C_22_ PUFAs) are synthesized from C_20_ PUFAs by action of elongase and desaturase [58]. C_2_-elongation of arachidonic acid (20:4n-6) forms docosatetraenoic acid (22:4n-6). The latter is converted to docosapentaenoic acid (22:5n-6) by Δ4 desaturase, which introduce a double bond in the FA chain at Δ4 position [59]. The same enzymes perform the synthesis of PUFAs of n-3 series: 20:5n-3 → 22:5n-3 → 22:6n-3 (from eicosapentaenoic acid to docosahexaenoic acid).

**Fig 5:**
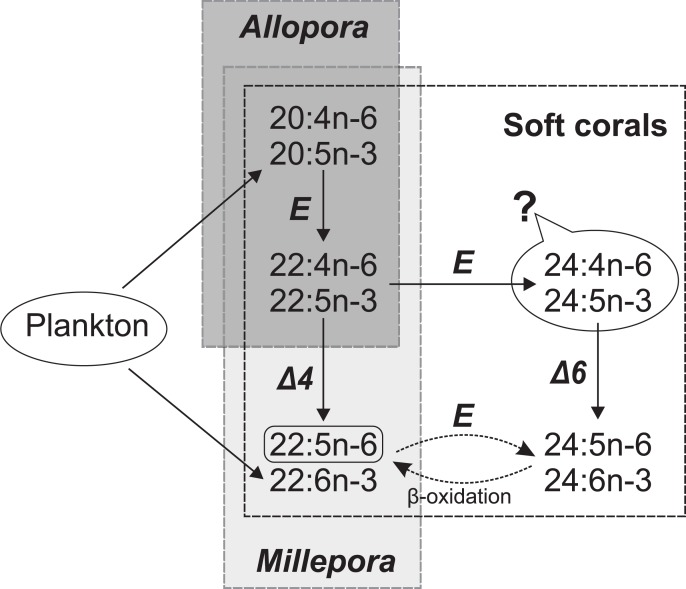
Possible pathways of biosynthesis of C_22-24_ PUFAs in soft corals and hydrocorals (*Allopora* and *Millepora*). E, Δ4, and Δ6 –C_2_ elongase, Δ4 desaturase, and Δ6 desaturase, respectively. 24:4n-6 and 24:5n-3 were not detected in total lipids of soft corals.

The existence of Δ4 desaturases has been discussed over the years. Acids 22:5n-6 and 22:6n-3 were found to be generated by Δ4 desaturation in primates, marine vertebrates, and lower eukaryotes [60], but no exact confirmation of the presence of Δ4 desaturase in cnidarians was reported. Sprecher [61] described an alternative pathway for C_22_ PUFAs biosynthesis, independent of Δ4 desaturase and involving two consecutive elongations and a Δ6 desaturation, which form C_24_ PUFAs, following by a two-carbon shortening via β-oxidation (for example, 20:5n-3 → 22:5n-3 → 24:5n-3 → 24:6n-3 → 22:6n-3). Sprecher’s pathway is possible for soft corals because they contain two C_24_ PUFAs, such as 24:5n-6 and 24:6n-3 [32] (Fig 5). This pathway requires the biosynthesis of 24:4n-6 and 24:5n-3, which are produced by one-step chain elongation of 22:4n-6 and 22:5n-3, respectively. However, with one exception (*Clavularia* sp. [62]), no intermediate products of this pathway (24:4n-6 and 24:5n-3) were detected in total FAs of soft corals [29,32]. Possible elongation of 22:5n-6 to 24:5n-6 and 22:6n-3 to 24:6n-3 in soft corals is, however, worth bearing in mind.

C_24_ PUFAs were not detected in any species of hydrocorals and hard corals [29,32], and therefore, these cnidarians cannot synthesize C_22_ PUFAs via Sprecher’s pathway. Food PUFAs and/or *de novo* biosynthesis via Δ4 desaturation may be the sources of 22:5n-6 and 22:6n-3 in these animals. The PUFA 22:6n-3 has been detected in corals and hydrocorals. [29,32]. Unfortunately, we cannot apply 22:6n-3 as an indicator of Δ4 desaturation of 22:5n-3, because the cnidarians consume plankton, being rich in 22:6n-3 [63]. On the other hand, 22:5n-6 is not obtained by cnidarians with food. Generally, only traces of 22:5n-6 were observed in total FAs of *Allopora steinegeri*, other cold-water hydrocoral species, cold-water soft corals, tropical hard corals, and tropical alcyonarians [29,32]. This observation shows a low activity or the absence of Δ4 desaturase in the cnidarian groups mentioned above (Fig 5). Total FAs of several species of tropical gorgonian soft corals contain up to 5% of 22:5n-6 [32], but, in this case, 22:5n-6 may theoretically originate from 24:5n-6 present in gorgonians.

The tropical *Millepora* species contained noticeable amount of 22:5n-6, which concentrated in structural GPL. We suppose that the high percentage of 22:5n-6 accompanied by the low percentages of 20:4n-6 and 22:4n-6 indicates a high activity of C_20_ → C_22_ elongase and Δ4 desaturase. The extremely high level of 22:6n-3 and the low level of 20:5n-3 confirm our hypothesis about the active elongase and Δ4 desaturase in *Millepora* tissues (Fig 5).

Overall, the distribution of C_20-24_ PUFAs in hydrocorals and corals is probably taxon-specific and does not depend on their habitat. This distribution seems to be explained by the presence of Δ4 desaturase in *Millepora* hydrocorals, the absence of this enzyme in reef-building corals and cold-water hydrocorals, and the activity of C_22_ → C_24_ elongase in soft corals. Direct experiments are nevertheless required to confirm the presence of Δ4 desaturase in *Millepora*.

### Distribution of PUFAs among glycerophospholipid classes

**Fig 6:**
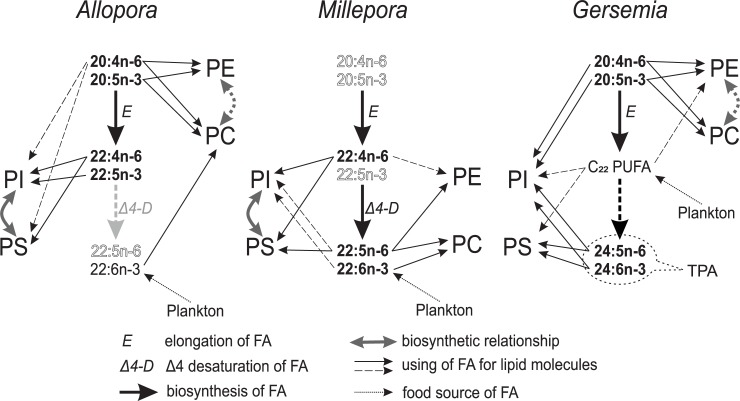
Distribution of C_20-24_ PUFAs among phospholipid classes of a soft coral and hydrocorals. Acyl groups of the molecular species of four phospholipid classes were compared between the cold-water hydrocoral *Allopora steinegeri*, tropical hydrocorals (*Millepora platyphylla*, *M*. *dichotoma*), and the cold-water soft coral *Gersemia rubiformis* [49]. Plankton are shown as a food source of 22:6n-3. Solid or transparent names of the polyunsaturated fatty acids (PUFAs) indicate high or low levels of these acids in total FAs, respectively. E, C_2_ elongase; Δ4, Δ4 desaturase, PE, ethanolamine glycerophospholipids; PC, choline glycerophospholipids; PS, serine glycerophospholipids; PI, inositol glycerophospholipids; TPA, tetracosapolyenoic acids.

A comparison of the hydrocorals studied with the cnidarians described earlier [18] (hard corals, soft corals, and hydrocorals) confirms that their polar lipids are comprised of four major phospholipids (PE, PC, PS, and PI) and one phosphonolipid (CAEP) irrespective of a taxonomic position and geographic region. Total lipids of zooxanthellate cnidarian species are known to include lipids of SDs. Glycolipids dominate the SD total lipids [47], whereas GPL are minor lipid classes, and CAEP is absent in SDs [64]. Hence, five polar lipid classes (PE, PC, PS, PI, and CAEP) mainly characterize animal (polyp) tissue of the cnidarians [32]. The polar lipidome of corals and hydrocorals was highly species-specific. A non-uniform distribution of C_20-24_ acyl groups among molecular species of different lipid classes was found. The general pathways of the PUFA distribution among GPL classes of some specimens of hydrocorals and soft corals are shown in Fig 6.

In tropical and cold-water alcyonarians (soft corals) PE and PC molecular species are mainly built on C_20_ PUFAs (20:4n-6 and 20:5n-3). PS molecular species is based on C_24_ acyl groups (24:5n-6 and 24:6n-3), whereas both C_20_ and C_24_ acyl groups are represented in PI molecular species [46,48,49] (Fig 6). Similar to alcyonarians, the cold-water *Allopora* hydrocoral builds its PE and PC molecular species on the basis of C_20_ PUFAs but synthesizes PI and PC using C_22_ PUFAs because of the lack of C_24_ PUFAs (Fig 6). In the unusual polar lipidome of the *Millepora*, all four GPL classes are mainly represented by molecules with C_22_ PUFA acyl groups (Fig 6). At the same time, the *Millepora* kept the difference in acyl composition between GPL classes: 22:5n-6 is directed to PE and PC, while 22:4n-6 is concentrated in PS and PI.

Temperature of gel-liquid transition of lipid bilayers is known to depend on the alkyl and acyl group composition of lipid molecules. An asymmetrical distribution of GPL classes between the internal and external sides of bilayer, as well as clustering of GPL, is characteristic of biological membranes [65]. It is possible that cnidarians regulate a local fluidity and some functions of biological membranes by selecting the appropriate PUFA acyl groups for each GPL class.

Along with phospholipids, CAEP has been characterized from a wide variety of marine invertebrates [66,67]. The key precursor of CAEP is 2-aminoethylphosphonate, which can be incorporated into lipid molecules by a pathway similar to that for PE biosynthesis [68]. A lot of PUFAs provide the diversity of GPL molecular species in alcyonarians and hydrocorals, whereas a few saturated FAs were detected in their CAEP. Compared to tropical *Millepora*, CAEP of cold-water *Allopora* contains considerable amounts of molecular species with short-chain acyl groups (14:0 and 15:0). The presence of short-chain acyl groups in lipid molecules is known to reduce the temperature of gel-liquid transition of lipid bilayers. The increase in the content of the structural lipids with short-chain acyl groups may be regarded as an adaptation of the *Allopora* to low environmental temperatures. It is possible that the higher level of CAEP with odd-chain groups, which are characteristic for bacteria lipids [69], is related with the odd-chain intermediates for CAEP synthesis obtained from an advance bacterial community or a bacterial food source of the *Allopora*. Instead of photosynthetic symbionts, associated bacteria were earlier suggested as a source of some FAs and lipids for the coral species without zooxanthellae [33,70].

### Relationships between the polar lipid classes

The comparison between soft corals and hydrocorals showed that most molecular species of PE and PC are comprised of the alkylacyl (plasmanyl) and alkenylacyl (plasmenyl) forms. According to the ether lipid biosynthesis pathways [71], 1-*O*-alkyl-2-acyl-*sn*-glycerols are utilized as substrates by choline- and ethanolaminephosphotransferases to form plasmanylcholines and plasmanylethanolamines, respectively, which are the alkyl analogs of phosphatidylcholine and phosphatidylethanolamine. The l'-alkyl desaturase system is responsible for the biosynthesis of ethanolamine plasmalogens from alkyl lipids. Only intact 1-*O*-alkyl-2-acyl-*sn*-glycero-3-phosphoethanolamine is known to serve as a substrate for the alkyl desaturase. Choline plasmalogens are probably derived from the ethanolamine plasmalogens.

These pathways of the ether lipid biosynthesis seem to be suitable to explain the PE and PC molecular species profiles observed in the chidarian species studied. The high similarity of the acyl groups among these two GPL classes confirms the same biosynthetic origin of most PE and PC molecular species. The selective action of the l'-alkyl desaturase explains the predominance of plasmalogens in PE molecular species compared to PC molecular species.

Another major pathway for the PE synthesis in eukaryotes is the decarboxylation of PS [65]. This reaction does not influence the non-polar parts of molecules, and, therefore, the possibility of head-group exchange between two GPL classes may be tested by a comparison of the non-polar parts of these classes. In soft corals, C_20_ PUFAs dominate in the PE acyl groups, while C_24_ PUFAs dominate in the PS acyl groups [46]. Hence, the decarboxylation of PS is not necessary for PE synthesis in soft corals. In hydrocorals, an asymmetrical resemblance between non-polar parts of PE and PS diacyl molecular species were detected. These data point on a possibility of head-group exchange between PE and PS in the hydrocorals.

Christie [54] emphasized that “the basic mechanism for biosynthesis of PS and PI is sometimes termed a branch point in phospholipid synthesis, as PE and PC are produced by a somewhat different route”. This statement is confirmed by the sharp difference in the alkyl/acyl composition between the PE/PC and PS/PI groups found in hydrocorals and soft corals. A prokaryotic-like pathway, when PS and PI are formed biosynthetically from phosphatidic acid via the intermediate cytidine diphosphate diacylglycerol (CDP-diacylglycerol) [65], may explain the high similarity of the alkyl/acyl composition between PS and PI molecular species in hydrocorals (S1 Fig). The concentration of the C_24_ PUFA acyl groups in PS and PI molecules also shows a biosynthetic relationship between these GPL classes in soft corals [46,48]. However, the pattern of this relationship in soft corals is not enough clear, because their PS is composed of alkylacyl molecules, whereas diacyl molecules dominate PI. The composition of the polar lipidomes indicates that soft corals have an additional way of PI biosynthesis, which does not include PS as an intermediate substrate.

In the present study, we described and compared the general images of the polar lipidome and the lipidomic fingerprints of several cnidarian taxa. We supposed that the basic features of the polar lipidomes of hydrocorals, reef-building corals and soft corals weakly depend on an influence of environmental factors and try to explain these features in lipid biosynthesis terms. Our study has shown that a comparative analysis of the polar lipidomes contributes to better understanding of the fatty acid biosynthetic pathways, the acyl and alkyl groups distribution among polar lipid classes, and the biosynthetic relationships between different phospholipid classes in marine invertebrates. We are sure that the lipidomic approach cans define more exactly the areas of purposeful investigations of marine lipid functions and biosynthesis.

## Supporting information

S1 Fig Comparison of mass spectra of two isomers of docosapentaenoic acid identified in the *Millepora* hydrocorals. The 4,4-dimethyloxazoline (DMOX) derivatives of (A) 4,7,10,13,16-docosapentaenoic acid (22:5n-6) and (B) 7,10,13,16,19-docosapentaenoic acid (22:5n-3) were analyzed by GC–MS. The structures of the derivatives and the predicted product ions are shown in each panel.
(DOCX)

S2 Fig Mass spectrometric fragmentation of ceramide aminoethylphosphonate (CAEP) of hydrocorals. MS/MS fragmentation patterns of positive ([M+H]^+^) and negative ([M−H]^−^) molecular ions exemplified by CAEP molecule with a long-chain 18:1 base (sphingosine) and 16:0 *N*-acyl group (18:1b/16:0 CAEP). This CAEP molecular species were found in the hydrocorals *Allopora steinegeri*, *Millepora dichotoma*, and *M*. *platyphylla*.
(DOCX)

S3 Fig Similarity of the acyl/alkylglycerol group compositions between the pairs of glycerophospholipid classes. The values against a gray background near each phospholipid class (in cycles) indicate the level of the molecular species (mol % of total molecular species of this class), which have the same acyl/alkylglycerol groups detected in the neighboring phospholipid class. PE, ethanolamine glycerophospholipids; PC, choline glycerophospholipids; PS, serine glycerophospholipids; PI, inositol glycerophospholipids.
(DOCX)

S1 Table Data underlying the findings described in the manuscript. The sheet “PUFAs”: The level of the major C20-22 polyunsaturated fatty acids (% of total acids, mean (AVG), and SD) obtained from total lipids and polar lipids of the hydrocorals *Allopora steinegeri*, *Millepora dichotoma*, and *M*. *platyphylla*. GC-MS chromatogram of DMOX derivatives of fatty acids of Millepora dichotoma is shown as an example. The sheet “Polar lipids”: Analysis of phospholipid and phosphonolipid composition (% of polar lipids) of three hydrocoral species with the examples of TLC plate and image analysis. The sheet “MS Millepora”: LC-MS analysis of the composition of polar lipid molecular species (retention times, *m/z* values, areas, and the percentage of each compound in polar lipid class and total polar lipids) of *Millepora* hydrocorals. The sheet “MS Allopora”: LC-MS analysis of the composition of polar lipid molecular species (retention times, *m/z* values, areas, and the percentage of each compound in polar lipid class and total polar lipids) of *Allopora* hydrocorals.
(XLSX)

S2 Table Lipid (% of total lipids) and phospholipid (% of polar lipids) compositions of three hydrocoral species. (DOCX)

S3 Table Fatty acid composition (%) of total lipids of three hydrocoral species. (DOCX)

S4 Table Composition of ceramide aminoethylphosphonate (CAEP) (mol % of polar lipids, mean ± SE, *n* = 3) of three hydrocoral species. (DOCX)
