# Supplementary material for: Comparative lipidomic analysis of phospholipids of hydrocorals and corals from tropical and cold-water regions
Source: PLoS One. 2019 Apr 29;14(4):e0215759. doi: 10.1371/journal.pone.0215759 (PMC6488065; doi:10.1371/journal.pone.0215759)
Supplement: S1 Fig — The 4,4-dimethyloxazoline (DMOX) derivatives of (A) 4,7,10,13,16-docosapentaenoic acid (22:5n-6) and (B) 7,10,13,16,19-docosapentaenoic acid (22:5n-3) were analyzed by GC–MS. The structures of the derivatives and the predicted product ions are shown in each panel. (DOCX) [file pone.0215759.s001.docx]

Comparative lipidomic analysis of phospholipid classes of hydrocorals and corals from tropical and cold-water regions

Andrey B. Imbs, Ly P. T. Dang, Kien B. Nguyen

**S1 Fig.** **Comparison of mass spectra of two isomers of docosapentaenoic acid identified in the *Millepora* hydrocorals.** The 4,4-dimethyloxazoline (DMOX) derivatives of (A) 4,7,10,13,16-docosapentaenoic acid (22:5n-6) and (B) 7,10,13,16,19-docosapentaenoic acid (22:5n-3) were analyzed by GC–MS. The structures of the derivatives and the predicted product ions are shown in each panel.

The positions of double bonds in the isomers were confirmed by mass spectrometry of their 4,4-dimethyloxazoline (DMOX) derivatives. Identical peaks of the molecular ions (M^+^, the mass-to-charge ratio (*m/z*) 383) were present in the mass spectra of DMOX derivatives of both isomers. The double bond in position 4 of the DMOX derivative of 4,7,10,13,16-docosapentaenoate (22:5n-6) was defined by the fingerprint ion at *m/z* 152 and the relative abundance of the ion at *m/z* 113 in comparison to that at *m/z* 126 (A). The gaps of 12 amu between *m/z* 166 and 178, 206 and 218, 246 and 258, 286 and 298 indicated the double bonds in positions 7, 10, 13, and 16, respectively. In contrast to the spectrum of 22:5n-6, the gaps of 12 amu between *m/z* 168 and 180, 208 and 220, 248 and 260, 288 and 300, 328 and 340 were found in the mass spectrum of the DMOX derivative of 7,10,13,16,19-docosapentaenoate (22:5n-3) (B).
