# Supplementary material for: Comparative lipidomic analysis of phospholipids of hydrocorals and corals from tropical and cold-water regions
Source: PLoS One. 2019 Apr 29;14(4):e0215759. doi: 10.1371/journal.pone.0215759 (PMC6488065; doi:10.1371/journal.pone.0215759)
Supplement: S2 Fig — MS/MS fragmentation patterns of positive ([M+H]+) and negative ([M−H]−) molecular ions exemplified by CAEP molecule with a long-chain 18:1 base (sphingosine) and 16:0 N-acyl group (18:1b/16:0 CAEP). This CAEP molecular species were found in the hydrocorals Allopora steinegeri, Millepora dichotoma, and M. platyphylla. (DOCX) [file pone.0215759.s002.docx]

Comparative lipidomic analysis of phospholipid classes of hydrocorals and corals from tropical and cold-water regions

Andrey B. Imbs, Ly P. T. Dang, Kien B. Nguyen

**S2 Fig.** **Mass spectrometric fragmentation of ceramide aminoethylphosphonate (CAEP) of hydrocorals.** MS/MS fragmentation patterns of positive ([M+H]^+^) and negative ([M−H]^−^) molecular ions exemplified by CAEP molecule with a long-chain 18:1 base (sphingosine) and 16:0 *N*-acyl group (18:1b/16:0 CAEP). This CAEP molecular species were found in the hydrocorals *Allopora steinegeri*, *Millepora dichotoma*, and *M. platyphylla*.

[M+H]^+^, [M+H+Et_3_N]^+^, [M+H−C_2_H_8_NO_3_P]^+^ ions, as well as [M−H]^−^ ions, present in the mass spectrum of each CAEP molecular species. In the MS^2^ spectra (positive mode), the [M+H]^+^ ions eliminated 2-aminoethylphosphonic acid (C_2_H_8_NO_3_P) or water (H_2_O) and formed two ions: [M+H−H_2_O]^+^ and [M+H−C_2_H_8_NO_3_P]^+^, whereas the [M+H−C_2_H_8_NO_3_P]^+^ ions eliminated water or dehydrated FA (keten) and formed two other ions: [M+H−C_2_H_8_NO_3_P−H_2_O]^+^ and [M+H−C_2_H_8_NO_3_P−keten]^+^ (Fig 3). In the MS^2^ spectrum (negative mode), the [M−H]^−^ ion lost the neutral keten and formed the main ion of aminoethylphosphonate of the long-chain base, which eliminated a water molecule in the MS^3^ spectrum. These characteristic ions allowed us to determine the numbers of carbon atoms and double bonds both in long-chain bases and in *N*-acyl groups of each CAEP molecular species.
