# Supplementary material for: Comparative lipidomic analysis of phospholipids of hydrocorals and corals from tropical and cold-water regions
Source: PLoS One. 2019 Apr 29;14(4):e0215759. doi: 10.1371/journal.pone.0215759 (PMC6488065; doi:10.1371/journal.pone.0215759)
Supplement: S3 Fig — The values against a gray background near each phospholipid class (in cycles) indicate the level of the molecular species (mol % of total molecular species of this class), which have the same acyl/alkylglycerol groups detected in the neighboring phospholipid class. PE, ethanolamine glycerophospholipids; PC, choline glycerophospholipids; PS, serine glycerophospholipids; PI, inositol glycerophospholipids. (DOCX) [file pone.0215759.s003.docx]

Comparative lipidomic analysis of phospholipid classes of hydrocorals and corals from tropical and cold-water regions

Andrey B. Imbs, Ly P. T. Dang, Kien B. Nguyen

**S3 Fig. Similarity of the acyl/alkylglycerol group compositions between the pairs of glycerophospholipid classes.** The values on a gray background near each phospholipid class (in cycles) indicate the content of the molecular species (mol % of total molecular species of this class), which have the same acyl/alkylglycerol groups detected in the neighbor phospholipid class. PE, ethanolamine glycerophospholipids; PC, choline glycerophospholipids; PS, serine glycerophospholipids; PI, inositol glycerophospholipids.
