# Supplementary material for: Comparative lipidomic analysis of phospholipids of hydrocorals and corals from tropical and cold-water regions
Source: PLoS One. 2019 Apr 29;14(4):e0215759. doi: 10.1371/journal.pone.0215759 (PMC6488065; doi:10.1371/journal.pone.0215759)
Supplement: S2 Table — (DOCX) [file pone.0215759.s005.docx]

Comparative lipidomic analysis of phospholipid classes of hydrocorals and corals from tropical and cold-water regions

Andrey B. Imbs, Ly P. T. Dang, Kien B. Nguyen

**S1 Table. Lipid (% of total lipids) and phospholipid (% of polar lipids) compositions of three hydrocoral species.**

| Lipid class | *Millepora platyphylla* | *Millepora dichotoma* | *Allopora steinegeri* |
| --- | --- | --- | --- |
| Wax esters (WE) | 37.1 | 26.7 | 10.5 |
| Monoalkyldiacylglycerols (MADAG) | 18.6 | 17.4 | 6.5 |
| Triacylglycerols (TG) | 22.0 | 22.8 | 36.8 |
| Free fatty acids (FFA) | 0.9 | 0.7 | 2.2 |
| Sterols (ST) | 5.4 | 5.6 | 17.4 |
| Polar lipids (PL) | 15.1 | 19.2 | 25.6 |
| Other lipids | 0.9 | 7.6 | 1.0 |
| Ethanolamine glycerophospholipids (PE) | 21.7 ± 0.7 | 21.1 ± 0.7 | 34.6 ± 1.2 |
| Choline glycerophospholipids (PC) | 39.7 ± 1.6 | 41.4 ± 1.0 | 39.4 ± 1.5 |
| Serine glycerophospholipids (PS) | 10.4 ± 1.0 | 9.9 ± 0.3 | 9.0 ± 1.7 |
| Inositol glycerophospholipids (PI) | 13.6 ± 0.7 | 11.8 ± 1.7 | 5.6 ± 0.7 |
| Ceramide aminoethylphosphonate (CAEP) | 14.7 ± 0.6 | 15.8 ± 0.4 | 11.7 ± 2.1 |
