# Supplementary material for: Comparative lipidomic analysis of phospholipids of hydrocorals and corals from tropical and cold-water regions
Source: PLoS One. 2019 Apr 29;14(4):e0215759. doi: 10.1371/journal.pone.0215759 (PMC6488065; doi:10.1371/journal.pone.0215759)
Supplement: S3 Table — (DOCX) [file pone.0215759.s006.docx]

Comparative lipidomic analysis of phospholipid classes of hydrocorals and corals from tropical and cold-water regions

Andrey B. Imbs, Ly P. T. Dang, Kien B. Nguyen

**S2 Table. Fatty acid composition (%) of total lipids of three hydrocoral species.**

| Fatty acid | Hydrocoral species |  |  |
| --- | --- | --- | --- |
|  | *Millepora platyphylla* | *Millepora dichotoma* | *Allopora steinegeri* |
| 14:0 | 3.1 | 2.3 | 2.5 |
| i-15:0 | - | - | 0.4 |
| 15:0 | 0.1 | 0.1 | 0.5 |
| 15:1 | - | 0.3 | - |
| i-16:0 | - | 0.3 | - |
| ai-16:0 | - | - | - |
| 16:0 | 23.6 | 19.8 | 12.7 |
| 16:1п-9 | 0.1 | 0.1 | 0.5 |
| 16:1n-7 | 0.1 | - | 1.8 |
| i-17:0 | - | - | 0.8 |
| ai-17:0 | - | - | 0.5 |
| 16:2n-7 | - | - | 0.2 |
| 7-Me-16:1n-10 | 0.5 | 0.4 | 0.5 |
| 17:0 | 0.2 | 0.1 | 0.5 |
| 17:1n-9 | - | - | 0.2 |
| 17:1n-7 | - | - | 0.6 |
| i-18:0 | - | 0.1 | 0.2 |
| 18:0 | 15.4 | 15.3 | 6.7 |
| 18:1n-9 | 6.1 | 3.9 | 4.8 |
| 18:1n-7 | 0.3 | 0.1 | 1.6 |
| 18:2n-6 | 0.5 | 0.1 | 1.6 |
| 19:0 |  | 0.2 | 0.4 |
| 18:3n-6 | 0.2 |  | 0.2 |
| 18:4n-3 | 1.5 | 1.9 | 0.7 |
| 20:0 | 3.3 | 5.5 | 0.4 |
| 20:1n-11 | - | - | 0.4 |
| 20:1n-9 | 0.2 | 0.4 | 8.4 |
| 20:1n-7 | - | - | 0.5 |
| 20:2n-6 | 0.3 | 0.1 | 0.4 |
| 20:3n-6 | 0.3 | 0.3 | 0.2 |
| 20:4n-6 | 0.7 | - | 17.5 |
| 20:3n-3 | - | - | 0.3 |
| 20:4n-3 | - | - | 1.3 |
| 20:5n-3 | 0.4 | 0.8 | 8.5 |
| 22:0 | 0.2 | 0.5 | - |
| 22:1n-9 | 0.2 | 0.3 | 0.5 |
| 22:1n-7 | - | - | 0.6 |
| 22:4n-6 | 2.6 | 3.5 | 4.7 |
| 22:5n-6 | 6.8 | 7.3 | 0.5 |
| 22:4n-3 | - | - | 0.5 |
| 22:5n-3 | 0.4 | 1.1 | 2.0 |
| 22:6n-3 | 32.0 | 33.3 | 13.3 |
| Other | 0.7 | 1.8 | 1.7 |
