# Supplementary material for: Comparative lipidomic analysis of phospholipids of hydrocorals and corals from tropical and cold-water regions
Source: PLoS One. 2019 Apr 29;14(4):e0215759. doi: 10.1371/journal.pone.0215759 (PMC6488065; doi:10.1371/journal.pone.0215759)
Supplement: S4 Table — (DOCX) [file pone.0215759.s007.docx]

Comparative lipidomic analysis of phospholipid classes of hydrocorals and corals from tropical and cold-water regions

Andrey B. Imbs, Ly P. T. Dang, Kien B. Nguyen

**S3 Table. Composition of ceramide aminoethylphosphonate (CAEP) (mol % of polar lipids, mean ± SE, *n* = 3) of three hydrocoral species.**

| Molecular species | [M–H]^–^, *m/z* detected | Content, mol %*^a^* | | |
| --- | --- | --- | --- | --- |
|  |  | *Allopora steinegeri* | *Millepora platyphylla* | *Millepora dichotoma* |
| 18:3b/14:0*^a^* | 611.4555 | 0.61 ± 0.08 | - | - |
| 18:2b/14:0 | 613.4638 | 1.71 ± 0.12 | - | - |
| 18:1b/14:0 | 615.4807 | 0.43 ± 0.02 | - | - |
| 18:0b/14:0 | 617.4938 | 2.95 ± 0.21 | - | - |
| 19:3b/14:0 | 625.4641 | 1.62 ± 0.13 | - | - |
| 18:3b/15:0 | 625.4641 | 0.11 ± 0.04 | - | - |
| 18:2b/15:0 | 627.4842 | 0.54 ± 0.05 | 0.15 ± 0.02 | 0.31 ± 0.01 |
| 18:1b/15:0 | 629.4987 | 0.09 ± 0.02 | - | - |
| 18:0b/15:0 | 631.5170 | 0.83 ± 0.03 | 0.02 ± 0.01 | 0.11 ± 0.04 |
| 20:3b/14:0 | 639.4754 | 0.11 ± 0.02 | - | - |
| 19:3b/15:0 | 639.4754 | 0.37 ± 0.01 | - | - |
| 18:2b/16:0 | 641.4962 | 0.45 ± 0.05 | 6.51 ± 0.44 | 6.38 ± 0.28 |
| 18:1b/16:0 | 643.5115 | 0.12 ± 0.00 | 5.86 ± 0.04 | 5.36 ± 0.24 |
| 18:0b/16:0 | 645.5311 | 0.31 ± 0.01 | 0.79 ± 0.34 | 1.74 ± 0.37 |
| 19:3b/16:0 | 653.4944 | 0.66 ± 0.04 | 0.14 ± 0.00 | 0.20 ± 0.16 |
| 18:2b/17:0 | 655.5098 | 0.33 ± 0.02 | 0.25 ± 0.03 | 0.42 ± 0.02 |
| 19:1b/16:0 | 657.5281 | - | 0.41 ± 0.11 | 0.62 ± 0.05 |
| 18:0 b/17:0 | 659.546 | - | 0.04 ± 0.03 | 0.27 ± 0.05 |
| 18:2b/18:0 | 669.5254 | 0.45 ± 0.07 | - | - |
| 18:1 b/18:0 | 671.5466 | - | 0.14 ± 0.02 | 0.09 ± 0.02 |
| 18:2 b/20:0 | 697.5621 | - | 0.15 ± 0.02 | 0.13 ± 0.05 |

*^a^*Structure description; sphingosine base / acyl chain
